# Comparison of CTAC and prone imaging for the detection of coronary artery disease using CZT SPECT

**DOI:** 10.1007/s12149-017-1194-z

**Published:** 2017-07-10

**Authors:** Shimpei Ito, Akihiro Endo, Taiji Okada, Taku Nakamura, Takashi Sugamori, Nobuyuki Takahashi, Hiroyuki Yoshitomi, Kazuaki Tanabe

**Affiliations:** 1 Division of Cardiology, Shimane University Faculty of Medicine, 89-1 Enya-cho, Izumo, 693-8501 Japan; 2 Clinical Laboratory Department, Shimane University Hospital, Izumo, Japan

**Keywords:** Single-photon emission computed tomography, Myocardial ischemia, Coronary artery disease

## Abstract

**Background:**

Cadmium-zinc-telluride (CZT) cameras have improved the evaluation of patients with chest pain. However, inferior/inferolateral attenuation artifacts similar to those seen with conventional Anger cameras persist. We added prone acquisitions and CT attenuation correction (CTAC) to the standard supine image acquisition and analyzed the resulting examinations.

**Methods and results:**

Seventy-two patients referred for invasive coronary angiography (CAG), and who also underwent rest/stress myocardial perfusion imaging (MPI) on a CZT camera in the supine and prone positions plus CTAC imaging, to examine known or suspected CAD between April 2013 and March 2014 were included. A sixteen-slice CT scan acquired on a SPECT/CT scanner between rest and stress imaging provided data for iterative reconstruction. Sensitivity, specificity, accuracy, and positive and negative likelihood ratios (LRs) were calculated to compare MPI with CAG on a per-patient basis. Per-patient sensitivity, specificity, and accuracy of supine images to predict coronary abnormalities on CAG were 35% [95% confidence interval (CI) 19–52], 86% (95% CI 80–92), and 74% (95% CI 66–82); those of prone imaging were 65% (95% CI 45–81), 82% (95% CI 76–87), and 78% (95% CI 68–85); and those of CTAC were 59% (95% CI 41–71), 93% (95% CI 87–97), and 85% (95% CI 76–91), respectively.

**Conclusions:**

Prone acquisition and CTAC images improve the ability to assess the inferior/inferolateral area.

## Introduction

Myocardial perfusion imaging (MPI) is useful for evaluating patients for coronary artery disease (CAD), assessing patients’ risk of future cardiac events, and evaluating therapeutic efficacy.

A recently developed high-efficiency ultrafast multi-pinhole cardiac camera with cadmium-zinc-telluride (CZT) detectors shows higher photon sensitivity and spatial resolution compared with conventional Anger cameras. Its overall accuracy has improved compared with the Anger camera [1, 2]. The increased sensitivity allows a lower dose and shorter image acquisition times [3].

Soft tissue attenuation of tracer activity, mainly in the inferior/inferolateral area, can result in artifactual perfusion abnormalities in the right coronary artery (RCA) and left circumflex (LCx) territories. The CZT camera, as with the Anger camera, displays these artifacts as inferior wall defects. As with the Anger camera [4–6] attempts to eliminate artifacts by imaging in the prone position have been reported [7–9]. Computed tomographic attenuation correction (CTAC) has also been reported to be a useful method to improve the specificity of a diagnosis of inferior wall ischemia [10, 11], and as for the use of a CZT camera, one study showed improvement of diagnostic accuracy with vascular territory analysis [12]. However, clinical experience using the CZT camera is limited, and there are few reports on using both prone acquisition and CTAC to reduce attenuation artifact, and no report has compared these measures employed as part of the same examination. We evaluated how addition of both prone imaging and CTAC might improve diagnostic accuracy in the inferior/inferolateral area.

## Methods

### Patients

The study population consisted of 85 consecutive patients who were referred for invasive coronary angiography (CAG), including planned routine follow-up after percutaneous coronary interventions (PCI), and also underwent rest/stress MPI on a CZT camera (Discovery NM 530c, GE Healthcare, Haifa, Israel) in the supine and prone positions and a 16-slice CT scan (Discovery NM/CT 670, GE Healthcare, Haifa, Israel) at Shimane University Hospital, Shimane, Japan, to examine known or suspected CAD between April 2013 and March 2014. Exclusion criteria were prior coronary artery bypass grafting (*n* = 4), hemodialysis (*n* = 3), and inability to lie prone (*n* = 6). The remaining 72 patients were investigated. This prospective study was approved by the Ethics Committee of Shimane University (No. 1242), and all patients provided written informed consent.

### Myocardial perfusion imaging protocol

**Fig. 1:**
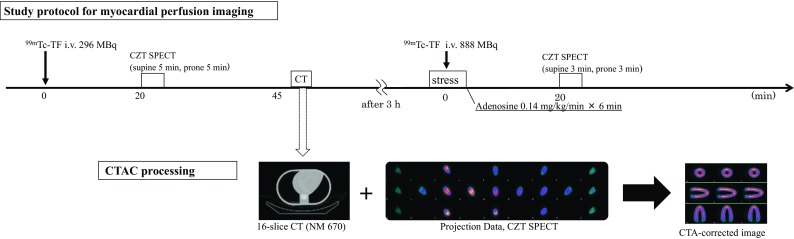
The combined MPI and CTAC protocol

All patients underwent 1-day rest/stress ^99m^Tc-tetrofosmin myocardial single-photon emission computed tomography (SPECT) pharmacologic protocol with adenosine (6-min infusion using a dose of 0.14 mg/kg/min) (Fig. 1). All patients were instructed to avoid caffeine-containing food and beverages for 14 h before the MPI study. The patients were injected with a dose of 296 MBq at rest and 888 MBq of ^99m^Tc-tetrofosmin during stress, in the arm opposite to the site of adenosine injection, after the first 3 min of adenosine infusion. The interval between the resting isotope infusion and stress radiopharmaceutical injections was 3 h. After a waiting period of 20 min after injection of the isotope, rest and stress images were acquired using the CZT camera in both the supine and prone positions with arms fully abducted, without any detector or collimator motion. All images were acquired with a 32 × 32 matrix and 20% energy window centered at the 140 keV photopeak of ^99m^Tc. Images were ECG-gated and acquired in list mode with a 5-min scan time for rest and a 3-min scan time for stress in each position. All SPECT images were reconstructed on a standard workstation (Xeleris Ver 3.0; GE Healthcare, Haifa, Israel).

### CT attenuation correction

An attenuation map was acquired on a Hybrid SPECT/CT system (Discovery NM/CT 670, GE Healthcare, Haifa, Israel) based on one CT scan in the supine position between rest and stress imaging. The CT image acquisition parameters were as follows: 120 kV, 10 mAs, field of view 500 mm, slice thickness 5 mm, image reconstruction filter SOFT, and adaptive statistical iterative reconstruction 40%.

### Myocardial perfusion image analysis

SPECT images were iteratively reconstructed using the transmission data generated from the 16-slice CT (Fig. 1). Reversible perfusion defects on MPI were assumed to be cardiac ischemia until CAG was performed. The findings of inferior/inferolateral wall ischemia on MPI were compared with the findings in the RCA or LCx on CAG.

Images were analyzed with commercially available software (Cedars QPS/QGS, Cedars-Sinai Medical Center, Los Angeles, CA, USA). Two experienced cardiologists performed quality analysis of myocardial perfusion images until CAG was performed. Reversible perfusion defects were visually diagnosed on MPI as cardiac ischemia. All fixed defects in this study were assumed to represent scar. We also performed semi-quantitative analysis using summed stressed score (SSS), summed rest score (SRS), and summed difference score (SDS). Cardiac ischemia was defined as a SDS ≥2 in each coronary territory. With respect to definition of inferior/inferolateral wall ischemia, the following American Heart Association (AHA) 17-segment model was used: LCx = segments 3, 4, 9, 10, and 15; RCA = segments 5, 6, 11, 12, and 16; and for anterior wall ischemia, LAD = segments 1, 2, 7, 8, 13, 14, and 17.

The findings of inferior/inferolateral wall ischemia on MPI were categorized as normal, ischemia (reversible defect), infarction (typical fixed defect), or equivocal (atypical fixed defect was classified as “normal without confidence”) based on the supine images. Changes in diagnosis of inferior/inferolateral wall ischemia after viewing prone and CTAC images as secondary validation were evaluated.

### Angiographic analysis

CAG was conducted following standard techniques. Caffeinated drinks and foods were discontinued for at least 14 h before the procedure. Cardiac ischemia was defined as a luminal narrowing of ≥90% by CAG using visual estimation. In all cases of intermediate stenosis (≥75%, ≤90%), fractional flow reserve (FFR) was measured and the presence of myocardial ischemia was determined when FFR <0.8.

### Statistical analysis

Continuous variables were expressed as mean ± SD, and categorical variables as percentages. Sensitivity, specificity, accuracy, and positive and negative likelihood ratios (LR) were calculated to predict the ability of MPI (each method) to identify myocardial ischemia in comparison with an ischemic result of CAG or FFR on a per-patient basis. Calculated 95% confidence intervals that were not overlapped were considered significant. All statistical analyses were performed using SPSS 19.0 (IBM, Armonk, NY, USA).

## Results

All patients successfully underwent rest/stress imaging with the CZT camera and invasive CAG one day after MPI. Between MPI and CAG, there were no signs of ischemia progression such as chest pain in any patient. The patient characteristics are shown in Table 1. Twenty-four patients (33%) were diagnosed with cardiac ischemia and 35 vessels were interpreted as abnormal with CAG and/or FFR. Two patients had triple-vessel disease. The number of patients with left anterior descending artery (LAD) stenosis, LCx stenosis, or RCA stenosis were 17 (24%), 14 (19%), and 9 (13%), respectively. Two patients had both LCx and RCA stenosis. There were no patients with left main trunk disease. Seventeen (24%) patients had LCx and/or RCA stenoses. Per-patient comparison of MPI with invasive CAG was assessed (Table 2). Visual per-patient analysis of MPI revealed reversible perfusion defects in 16 patients (22%) with standard supine images, 23 patients (32%) with prone images, and 15 patients (21%) with CTAC images. Per-patient sensitivity, specificity, and accuracy of supine images to predict cardiac ischemia on CAG were 35% [95% confidence interval (CI) 19–52], 86% (95% CI 80–92), and 74% (95% CI 66–82); those of prone images were 65% (95% CI 45–81), 82% (95% CI 76–87), and 78% (95% CI 68–85), and those of CTAC images were 59% (95% CI 41–71), 93% (95% CI 87–97), and 85% (95% CI 76–91), respectively. Positive LRs were 2.4 (95% CI 1.6–3.7) in supine, 3.6 (95% CI 2.8–4.4) in prone, and 8.1 (95% CI 4.7–13.8) in CTAC images, showing significant differences between supine and CTAC images to predict cardiac ischemia. Negative LR s were 0.76 (95% CI 0.71–0.81) in supine, 0.43 (95% CI 0.35–0.54) in prone, and 0.44 (95% CI 0.38–0.53) in CTAC image, showing significant difference between supine images and other two images. According to semi-quantitative analysis, per-patient sensitivity, specificity, and accuracy of supine images to predict cardiac ischemia on CAG were 24% (95% CI 11–38), 91% (95% CI 87–95), and 75% (95% CI 69–83); those of prone images were 35% (95% CI 19–50), 91% (95% CI 86–95), and 78% (95% CI 70–85), and those of CTAC images were 35% (95% CI 19–52), 87% (95% CI 82–92), and 75% (95%CI 67–83), respectively. Thus, results based on a semi-quantitative analysis were inferior to those based on quality analysis.

**Table 1:** Patient characteristic

Characteristic	Value
Male, *n* (%)	58 (81)
Age (years)	72 ± 9
BMI (kg/m^2^)	24 ± 4
Cardiovascular risk factor, *n* (%)	
Obesity (BMI >30 kg/m^2^)	6 (8)
Diabetes mellitus	28 (39)
Smoking	47 (65)
Hypertension	55 (76)
Dyslipidemia	40 (56)
Positive family history	7 (10)
Clinical symptoms, *n* (%)	
Typical angina pectoris	13 (18)
Atypical chest pain	11 (15)
Dyspnea on exertion	18 (25)
No cardiac symptoms	30 (42)
Previous cardiac events, *n* (%)	
Myocardial infarction	22 (31)
Percutaneous coronary intervention	38 (53)
Current cardiac medication, *n* (%)	
Aspirin	50 (69)
Clopidogrel	37 (51)
Beta-blocker	32 (44)
ACE/angiotensin II inhibitor	44 (61)
Statin	49 (68)

Data presented as mean ± SD or *n* (%)

*BMI* body mass index, *ACE* angiotensin-converting enzyme

**Table 2:** Per-patient comparison of MPI to invasive CAG

	Sensitivity, % (95% CI)	Specificity, % (95% CI)	Accuracy, % (95% CI)	Positive LR (95% CI)	Negative LR (95% CI)
Supine	35 (19–52)	86 (80–92)	74 (66–82)	2.4 (1.6–3.7)	0.76 (0.71–0.81)
Prone	65 (45–81)	82 (76–87)	78 (68–85)	3.6 (2.8–4.4)	0.43 (0.35–0.54)
CTAC	59 (41–71)	93 (87–97)	85 (76–91)	8.1 (4.7–13.8)	0.44 (0.38–0.53)

*95% CI* 95% confidence interval, *CTAC* CT attenuation correction, *LR* likelihood ratio

**Fig. 2:**
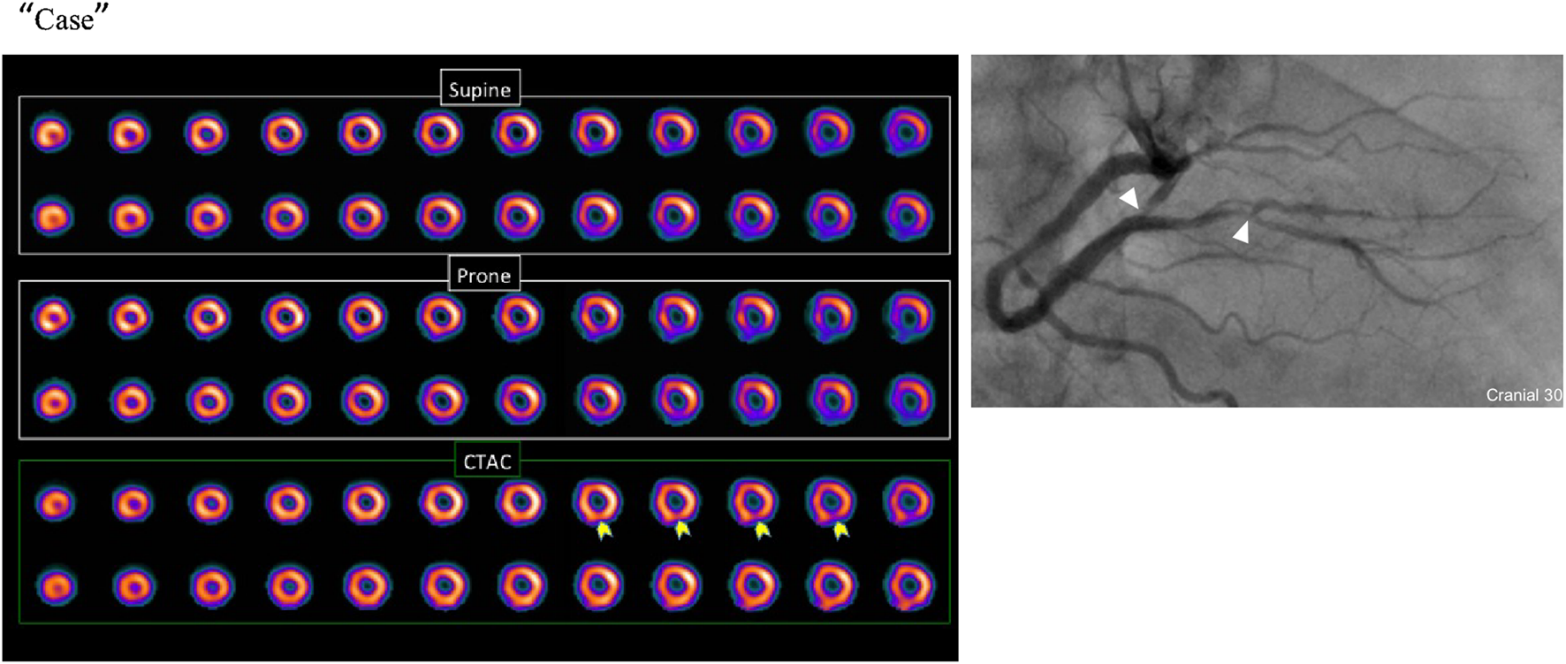
A 72-year-old man experiencing chest pain. CTAC images elucidated inferior ischemia that could not be seen on supine or prone images. CAG showed 90% stenosis of #4PL

We present a case in which the CTAC image was more useful for diagnosing inferior wall ischemia than the supine and prone images. A 72-year-old man experienced chest pain, and cardiac catheterization was planned. MPI was performed the day before CAG. CTAC images showed inferior ischemia that could not be seen on the supine and prone images. CAG showed 90% stenosis of #4PL (Fig. 2).

FFR was measured in the RCA or LCx in 12 patients because of intermediate coronary artery stenosis. Three patients were positive; the other nine patients, including one triple-vessel disease (TVD) patient, were negative. With respect to FFR-positive patients, all images were positive in MPI in one patient, but all images were negative in the other patients. In regard to FFR-negative patients, four patients were positive in supine images, while in prone and CTAC images one patient with triple-vessel disease was positive, and the others were negative.

On the other hand, diagnostic evaluation for anterior wall ischemia was as follows: sensitivity, specificity, and accuracy with visual estimation were 55% (95% CI 33–77), 82% (95% CI 78–86), and 78% (95% CI 70–85); those of semi-quantitative analysis were 73% (95% CI 47–90), 85% (95% CI 81–88), and 83% (95% CI 75–89), respectively.

### Secondary validation

**Fig. 3:**
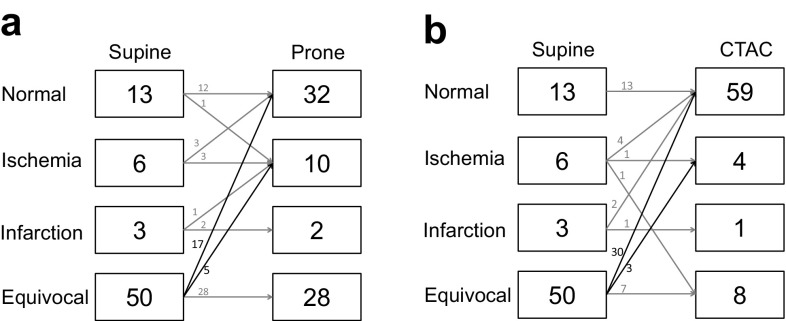
Changes in diagnosis when adding prone (**a**) and CTAC (**b**) images in each case

We evaluated how a diagnosis based on supine images changed when adding prone and CTAC images in each case (Fig. 3a, b).

Forty cases judged to be equivocal based on supine images decreased to 28 cases on prone images and eight cases on CTAC images. There were 17 cases in which diagnosis varied from equivocal to normal in prone images, and 30 cases in which diagnosis varied from equivocal to normal in CTAC images. There were five cases that varied from an equivocal diagnosis in the supine images to an ischemia diagnosis in the prone images. Three of these cases were shown to have cardiac ischemia by catheterization. Likewise from the supine position images to CTAC images, the diagnosis of three cases changed from equivocal to ischemia. All three cases were shown to have myocardial ischemia by catheterization.

## Discussion

We analyzed the diagnostic accuracy for inferolateral wall ischemia on MPI in the prone position and with CTAC and the usual supine position images individually, in comparison with CAG performed the next day. There are several reports that compare CZT MPI with invasive CAG [8, 13, 14]. However, the interval between the two examinations in these studies was 2–3 months, while in our study it was only 1 day. No ischemic symptoms occurred in the patients between the examinations.

In this study, adding prone and CTAC images had favorable outcomes in terms of the ability to diagnose inferior/inferolateral wall ischemia. In particular, CTAC images showed significantly better performance to predict cardiac ischemia based on inferior/inferolateral wall ischemia compared usual supine images. Use of both prone and CTAC images decreased the number of inferior/inferolateral equivocal interpretations.

Nuclear cardiology is one of the most useful tests in the stratification of risk for cardiac events and treatment assessment. MPI is a useful examination to determine that patients will likely benefit from invasive CAG followed by coronary revascularization, which may be well suited to medical therapy, and which are not suffering from CAD [15]. Recently, the new CZT cameras have made a significant difference in evaluation of patients for CAD. However, troublesome artifact effects of the inferior wall exist similarly to those on images obtained with conventional Anger cameras. There is a possibility that the arrangement of the 19 pinhole collimators of the CZT camera may be more sensitive to the presence of hepatic and bowel activity compared with the standard Anger camera with a 360° arc acquisition [16].

The use of prone imaging can reduce diaphragmatic attenuation and improve inferior wall image quality. The rationale for prone imaging is that the heart shifts slightly superiorly and the diaphragm is more inferior in the prone position, increasing the distance between the diaphragm and the inferior wall of the left ventricle. It is said to be useful to add a prone image to diagnose inferior wall ischemia with a conventional camera [4–6] and a similar report has been published for the CZT camera [7–9]. Our study also shows the improvement in diagnostic performance for inferior lateral wall ischemia.

Attenuation correction refers to automated methods in which the intensity of the myocardial perfusion image is adjusted to reflect the estimated magnitude of soft tissue attenuation on different regions of the heart, resulting in improved SPECT MPI diagnostic [10] and prognostic [11] accuracy. Attenuation correction methods include either line-source or CT techniques. CTAC correction is reported to be one of the most useful methods to solve the artifactual problems in the inferior wall that appear in images obtained with Anger cameras. With respect to Discovery NM 530c, a study using this method was recently reported by Emory University [17]. Several studies with respect to utility of CTAC in CZT have been conducted. Caoballi et al. assessed 44 patients comparing MPI in stress-first with CZT camera using CTAC and CAG within 6 months, and they showed CTAC improved diagnostic accuracy by improving specificity over uncorrected images [12]. We also assessed the diagnostic quality of images using CT-based transmission data generated with 16-slice CT for iterative reconstruction of data acquired with the CZT camera. This method significantly enhanced the ability to diagnose inferior/inferolateral wall ischemia.

In our study, the addition of prone images or CTAC images turned out to be useful. In addition, this was the first report in which the diagnostic quality of images based on prone images and CTAC, in addition to simple supine images, obtained with the CZT camera was estimated. It was clear that the diagnostic quality was better than the supine images alone for detecting inferior/inferolateral wall ischemia. The lower dose used with the CZT camera allows a lower radiation dose to the patient and faster throughput. Further studies are warranted to develop a database of normal prone and CTAC images as this method becomes commonplace.

The reported overall sensitivity of vasodilator stress perfusion SPECT for the detection of angiographically significant (≥50% stenosis) CAD is 87%, and specificity is 73% [18]. Although our analysis was limited to the inferior/inferolateral area, the sensitivity was poor. We were not able to determine clearly whether an image on MPI indicated ischemia or not, because of the problem of artifact in the inferior/inferolateral area. It is important in assessing a moderately fixed defect that we cannot consider “equivocal” as normal in clinical practice. The percentage of equivocal interpretations of supine images in this study was higher (69%) than that reported in other studies (approximately 20–40%) [19, 20]. We also assessed how these equivocal findings changed when prone and CTAC images were added. Equivocal interpretations decreased to 39 and 11% when using prone and CTAC images, respectively. Three of 5 cases of prone imaging and all three cases of CTAC images that varied from equivocal to ischemia in supine images corresponded to significant stenosis on catheterization. These changes led to an increase in sensitivity. These changes are the main point in emphasizing the use of adding prone and CTAC images.

If the use of CTAC is established, prone imaging may not always be needed in assessing the inferior/inferolateral area, and examination time will be reduced. Another advantage of CTAC imaging is that it can perform well in elderly people with kyphosis, who may have difficulty assuming the prone position. Aging is universal, and it seems that the use of assessment of the inferior/inferolateral area by CTAC imaging will increase. Cardiac CT is used widely to assess for coronary artery disease. In cases where CT images were recently obtained, we hope that these images will be useful for CTAC. We expect this to offer improvement over the simple supine position protocol in artifact problems of the inferior/inferolateral area. The radiation exposure for CTAC is much lower than that for routine chest CT. The radiation dose of a conventional chest CT is approximately 20 mGy (CT dose index; CTDI). The CTDI for the CT attenuation acquisition is 0.68 mGy.

Studies comparing CAG and high-efficiency CZT camera SPECT detecting CAD have been reported. Few studies have been reported with respect to assessment of diagnostic performance of a CZT camera using FFR [21, 22]. Tanaka et al. reported that with addition of prone imaging, CZT SPECT had a high diagnostic yield in detecting significant coronary stenosis as assessed using FFR [21]. However, there are no studies regarding the addition of both prone and CTAC imaging. FFR is another well-established index for investigating the physiological significance of a coronary stenosis. Large clinical trials such as the FAME trial have also adopted the more inclusive FFR threshold of 0.80 in main epicardial vessels [23]. We assessed cardiac ischemia based on FFR in all cases where intermediate stenosis was confirmed by CAG. There have been few CZT MPI studies based on a “gold standard” for the diagnosis of CAD, which may involve both anatomical and functional assessments such as FFR, positron emission tomography, or magnetic resonance imaging. Assessments with FFR enhance the value of the present study to the extent of performing FFR to all branches with intermediate stenosis.

## Limitations

This study was limited by the single-site clinical experience, and by a small study population. The study included a mixed population of patients, including those in whom CAG was performed as follow-up. Furthermore, there were two cases of triple-vessel disease, and it was thought that there is a limit in the diagnosis of multivessel disease based on images obtained using the CZT camera. In such cases, where there is widespread myocardial ischemia, perfusion imaging techniques preferentially identify only the myocardial perfusion defect in the most ischemic territory. The timing of injection was relatively early in our protocol, and thus effects from the liver were rather strong. This problem may have decreased sensitivity in the supine position. In most cases where we were unable to diagnose ischemia, there were effects of hepatic accumulation. We often encountered soft tissue attenuation due to increased radionuclide tracer in the liver. Further examination of the timing of the imaging after injection is needed, and no gated MPI analysis was conducted, which may have helped differentiate between artifacts and infarction in this study.

## Conclusion

Adding prone acquisition and CTAC–corrected supine images improved the ability to identify inferior/inferolateral area defects.
